# BOSC 2021, the 22nd Annual Bioinformatics Open Source Conference

**DOI:** 10.12688/f1000research.74074.1

**Published:** 2021-10-18

**Authors:** Nomi L. Harris, Peter J. A. Cock, Christopher J. Fields, Karsten Hokamp, Jessica Maia, Monica Munoz-Torres, Malvika Sharan, Jason Williams

**Affiliations:** 1 Environmental Genomics and Systems Biology, Lawrence Berkeley National Laboratory, Berkeley, CA, 94720, USA; 2 Information and Computational Sciences, The James Hutton Institute, Dundee, DD2 5DA, UK; 3 Carver Biotechnology Center, University of Illinois Urbana-Champaign, Urbana, IL, 61801, USA; 4 Smurfit Institute of Genetics, Trinity College Dublin, Dublin 2, D02 PN40, Ireland; 5 BD, Durham, NC, 22709, USA; 6 Biochemistry and Molecular Genetics Department, University of Colorado School of Medicine, Aurora, CO, 80045, USA; 7 The Alan Turing Institute, London, NW1 2DB, UK; 8 Cold Spring Harbor Laboratory, Cold Spring Harbor, NY, 11724, USA

**Keywords:** bioinformatics, open source, open science, online conference

## Abstract

The 22nd annual Bioinformatics Open Source Conference (BOSC 2021, open-bio.org/events/bosc-2021/) was held online as a track of the 2021 Intelligent Systems for Molecular Biology / European Conference on Computational Biology (ISMB/ECCB) conference. Launched in 2000 and held every year since, BOSC is the premier meeting covering topics related to open source software and open science in bioinformatics. In 2020, BOSC partnered with the Galaxy Community Conference to form the Bioinformatics Community Conference (BCC2020); that was the first BOSC to be held online.

This year, BOSC returned to its roots as part of ISMB/ECCB 2021. As in 2020, the Covid-19 pandemic made it impossible to hold the conference in person, so ISMB/ECCB 2021 took place as an online meeting attended by over 2000 people from 79 countries. Nearly 200 people participated in BOSC sessions, which included 27 talks reviewed and selected from submitted abstracts, and three invited keynote talks representing a range of global perspectives on the role of open science and open source in driving research and inclusivity in the biosciences, one of which was presented in French with English subtitles.

## Introduction

First held in 2000, as part of Intelligent Systems for Molecular Biology (ISMB), the Bioinformatics Open Source Conference (BOSC) has been part of ISMB every year since, except for 2018 and 2020, when we partnered with the Galaxy Community Conference (GCC). For 2021, we returned to our roots and held BOSC again as part of ISMB/European Conference on Computational Biology (ECCB) 2021.

Originally planned for Lyon, France, ISMB/ECCB 2021 was switched to an online event due to Covid-19. As we learned from our experiences in 2020, an online meeting has pros and cons. On the plus side, registration fees are generally lower than for comparable in-person events, and people from all over the world are able to attend without any travel requirement. On the minus side, a virtual event typically can’t recreate the opportunities for social interactions and serendipitous encounters that are among the most valuable aspects of an in-person meeting.

ISMB/ECCB 2021 was hosted by the Showcare event service, which provides an integrated platform with a layer over Zoom. In addition, many BOSC attendees used our public Slack workspace for conversation before, during, and after the meeting.

### Accessibility and inclusion

In 2021, we continued our efforts to advance and strengthen diversity and inclusion within BOSC. Some of the strategies we implemented this year include:

**Free registration.** Recognizing that the registration fee for ISMB/ECCB (which includes more than a dozen different tracks in addition to BOSC) could be a barrier to some, we offered multiple opportunities for interested participants to request free registration. Participants submitting abstracts could request assistance to cover their registration fee by checking a box on the submission form. This year, we announced a special, third round of OBF Event Fellowships (usually offered twice a year) focused on supporting potential BOSC participants who had not submitted abstracts. OBF Event Fellowships aim to increase participation from people who identify with groups that are traditionally underrepresented in bioinformatics and open science events.

**Figure 1.**
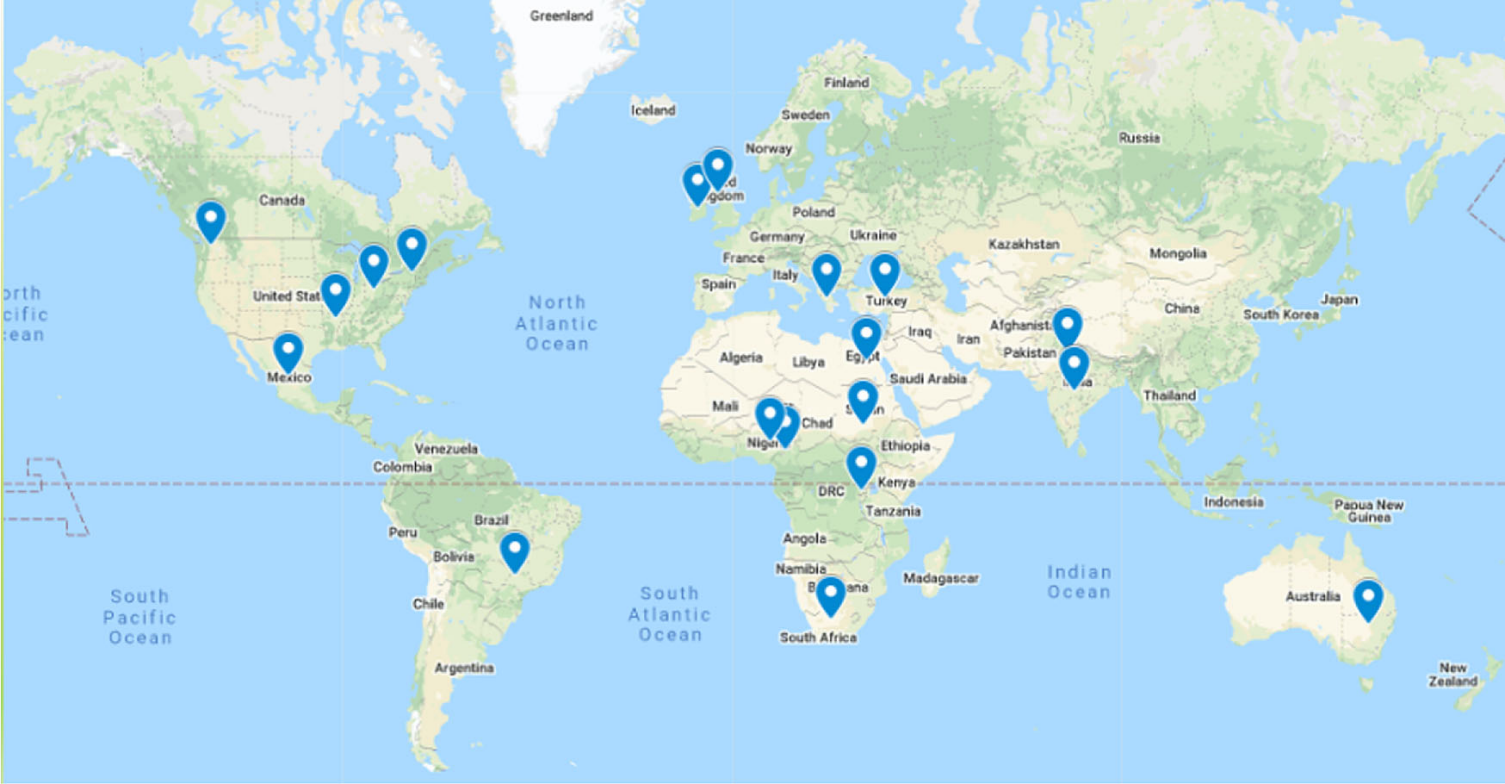
Locations of Bioinformatics Open Source Conference participants who received free registration to Intelligent Systems for Molecular Biology/European Conference on Computational Biology 2021.

Thanks to our sponsors, we were able to grant free registration to everyone who asked for assistance, a total of 20 people from around the world (see Figure 1), not including organizing committee members and gold/platinum sponsor representatives whose registration fees were also paid for. You can read blog posts by some of the OBF/BOSC Event Fellowship Awardees on our website: https://www.open-bio.org/blog/.

**Honoraria.** This year, for the first time, we offered honoraria to our keynote speakers. Those who did not want to accept honoraria were given the option to donate it back to BOSC towards supporting future participants in need. We recognize that working on open source projects often involves contributing effort that is uncompensated. At the same time, individuals from communities that have been historically excluded from science and open source communities often face higher burdens to participation, and therefore less time to give as volunteer effort. Therefore, we made a decision to offer honoraria in recognition of the fact that not all researchers are privileged to be able to gift their time.

**Including non-English-speaking presenters.** While English is predominantly considered the default language for communicating research findings, most of the world’s scientists speak it as a second language. The assumption that it is the international language of science can potentially create a huge barrier to participation and contributions from many research communities around the world. Thomas Hervé Mboa Nkoudou, a leader in the biotech maker movement in Africa, delivered his keynote talk for BOSC in French with English subtitles (see Keynotes section below). We believe this to be the first talk to be presented at ISMB in a language other than English. The response to this bilingual keynote talk was quite positive, and we hope this will help pave the way to including the voices of more researchers across the globe in ways that are inclusive of communities who don’t use English as their first language.

**Videos.** For those who were not able to attend BOSC 2021, we made all the talk videos available immediately after the conference on our YouTube channel: https://bit.ly/BOSC2021-YouTube. (The poster videos were added to our channel about a month later.) In addition to enabling the talks to be viewed any time, without requiring conference registration, the YouTube videos have the advantage of optional computer-generated closed captioning.

## Conference program

### Keynotes

**Figure 2.**
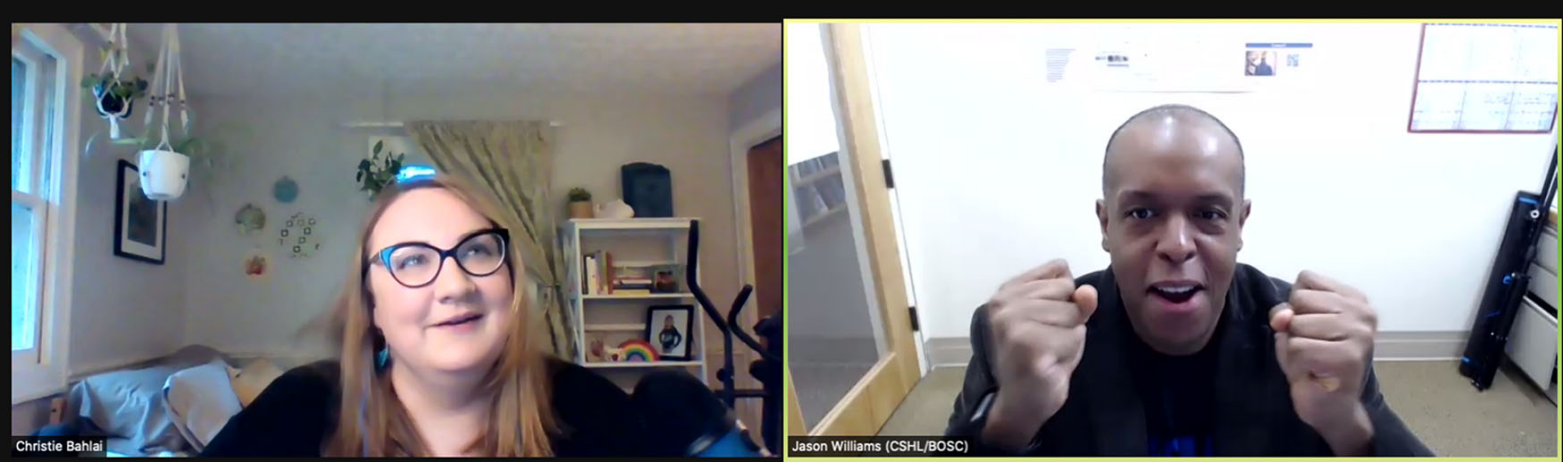
Keynote speaker Christie Bahlai is cheered on by moderator Jason Williams.

The first BOSC 2021 keynote talk, “Significant heterogeneities: Ecology’s emergence as open and synthetic science”, was delivered live by Christie Bahlai ( Figure 2). She started by acknowledging that her talk is a highly subjective personal journey, and since interaction between technology, culture, and science shapes our career path, it's really important to put them in the context of our personal stories and reflect on them. Ecology has not been a big topic at past BOSCs, and Christie’s approach to open and inclusive science strongly resonated with our community. One attendee commented, “Christie Bahlai's keynote was inspiring and a breath of fresh air. +1 to calling out ‘open science purists’ and the call for collective responsibility.”

**Figure 3.**
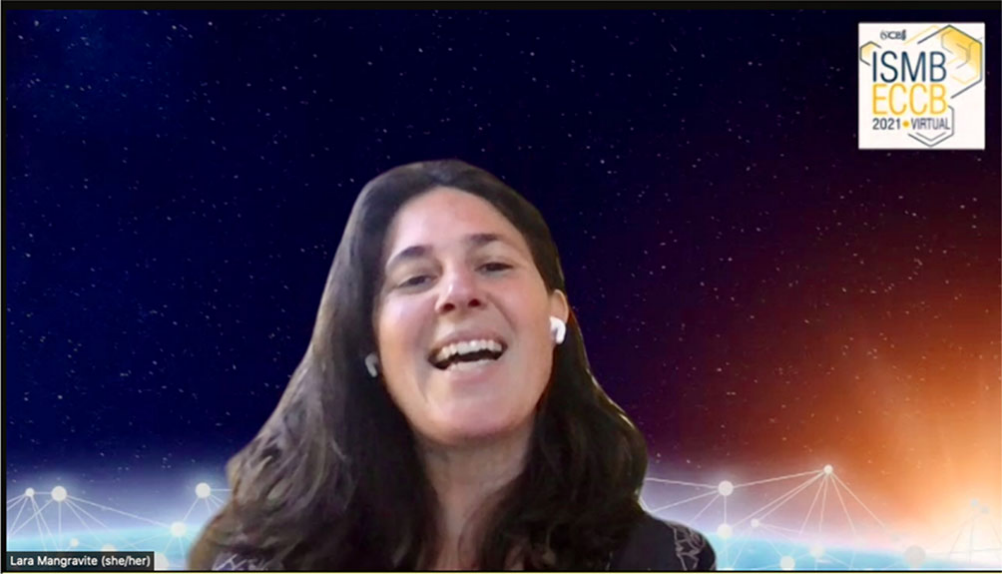
Lara Mangravite was the keynote speaker in a joint Bioinformatics Open Source Conference (BOSC) /Function Community of Special Interest (COSI) session.

Our second keynote, by Lara Mangravite ( Figure 3), was part of a joint session with the Function Community of Special Interest (COSI), chaired by Iddo Friedberg. Lara spoke on “Open approaches to advance data-intensive biomedicine.” Over 200 participants attended this joint session. Lara noted that clinical applications need access to high-quality data, but broad accessibility of human clinical data is difficult due to privacy issues, and access to analysis capabilities is distributed inequitably and tends to leave out those in the global south. She discussed some possible mitigations for those challenges.

**Figure 4.**
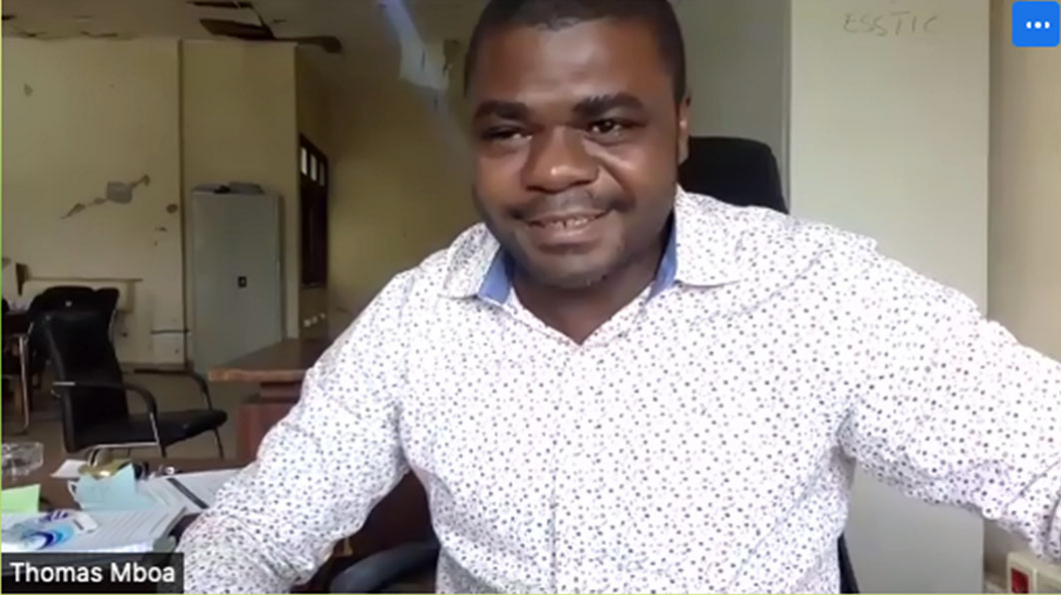
Keynote speaker Thomas Mboa answered questions from his office in Cameroon.

The final keynote speaker at BOSC 2021 was Thomas Hervé Mboa Nkoudou of Cameroon ( Figure 4), whose talk title was “Contribution of the maker movement to biotechnology in Africa: An open science perspective”. This was the first BOSC talk not given in English; instead, Thomas spoke in French with English subtitles. Thomas emphasized that open source/open data are key to wide dissemination of knowledge in the biosciences and beyond. An example of this openness in practice is the way the maker movement, which embraces openness, has contributed to the democratization of biotechnology in Africa. Thomas observed that universities have not yet embraced openness and the maker-movement philosophy in African countries; an attendee noted that this is also the case in Europe. Thomas responded that to address this, we need to teach principles, philosophy and give practical experience as early as possible so that new generations consider maker/DIYbio solutions when they are faced with problems themselves.

### Talks

As usual, BOSC included a smorgasbord of talks chosen from submitted abstracts. The 2021 talk schedule can be found here, and the session topics were:

• Standards and Practices for Open Science
• Tools for Open Science
• Joint session with Function COSI
• Analysis tools
• Workflow Management Systems
• Visualization
• Translational Bioinformatics

**Figure 5.**
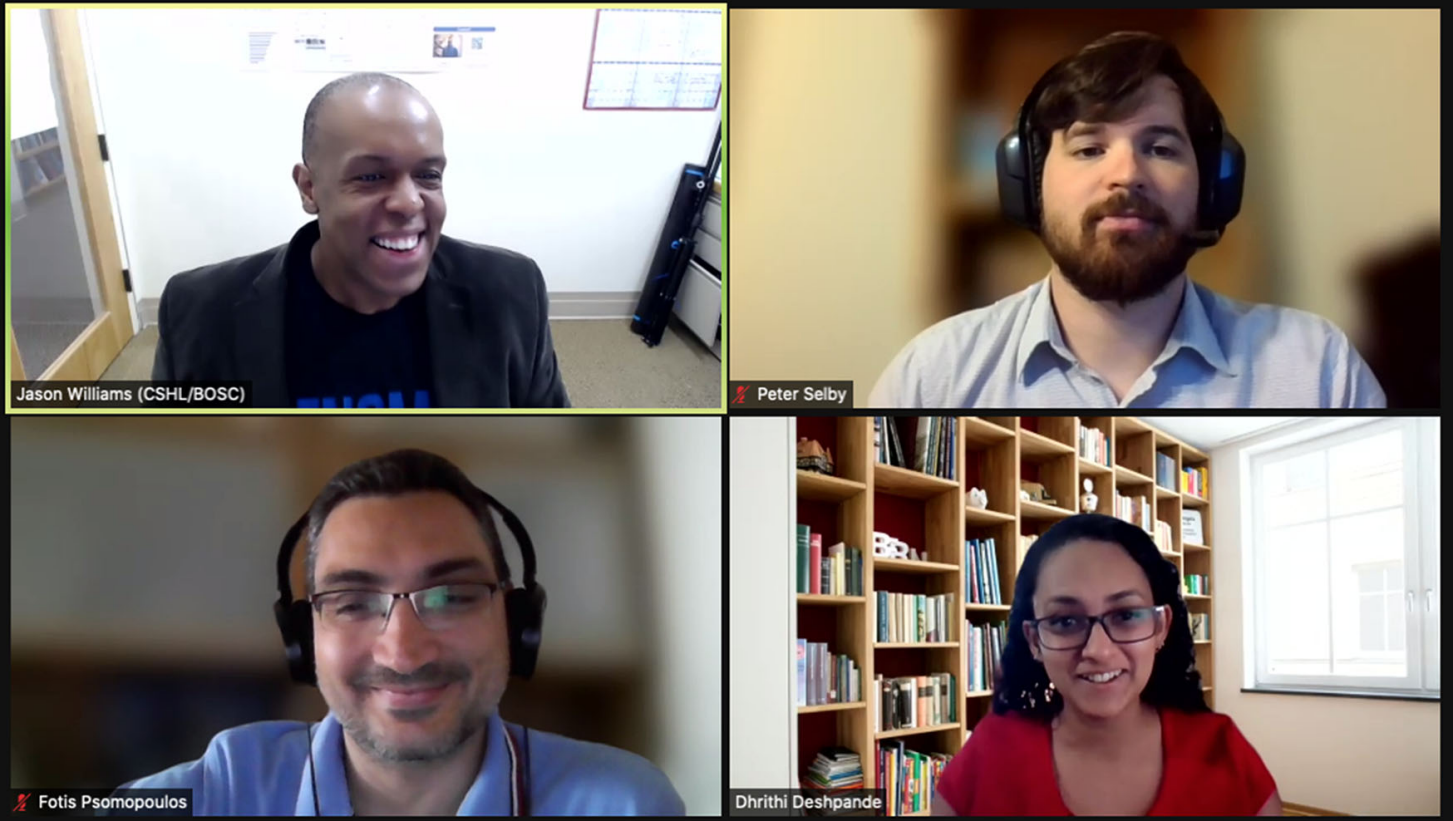
Session chair Jason Williams and some of the presenters in the Standards and Practices for Open Science session.

**Figure 6.**
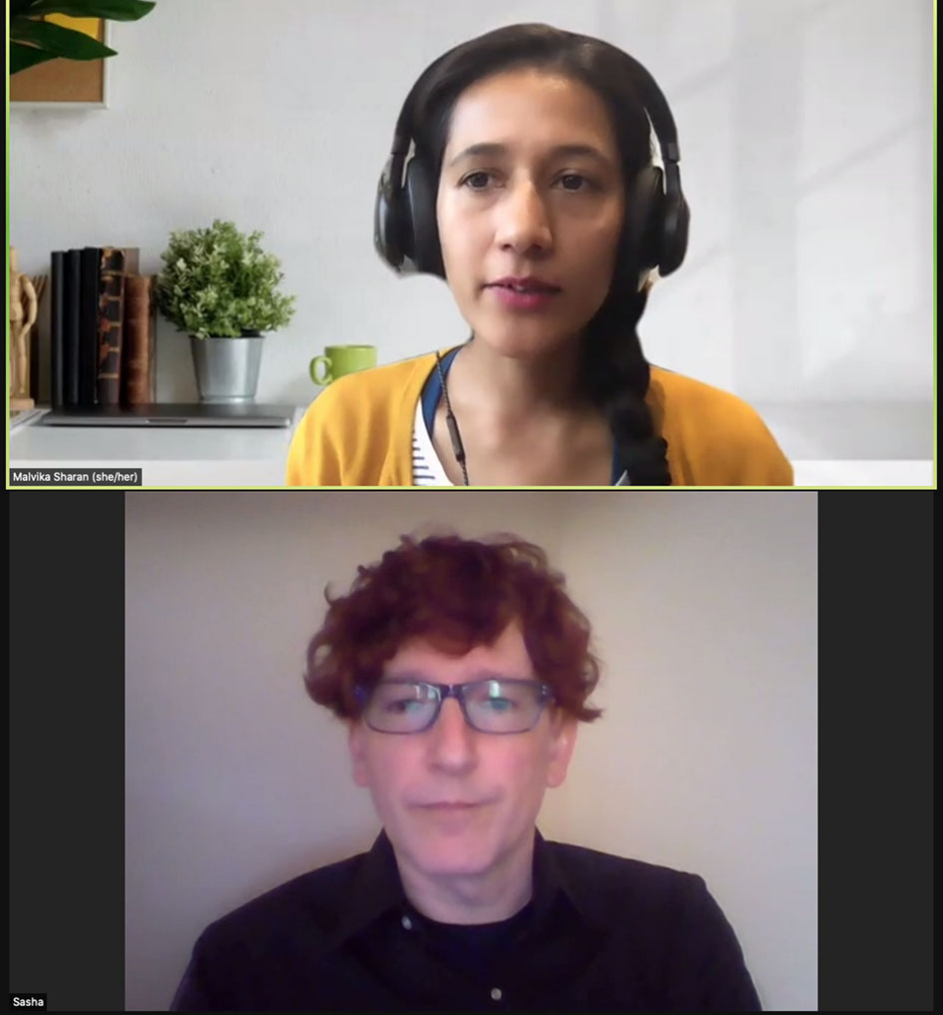
“Tools for Open Science” session chair Malvika Sharan moderates questions for presenter Sasha Zaranek.

**Figure 7.**
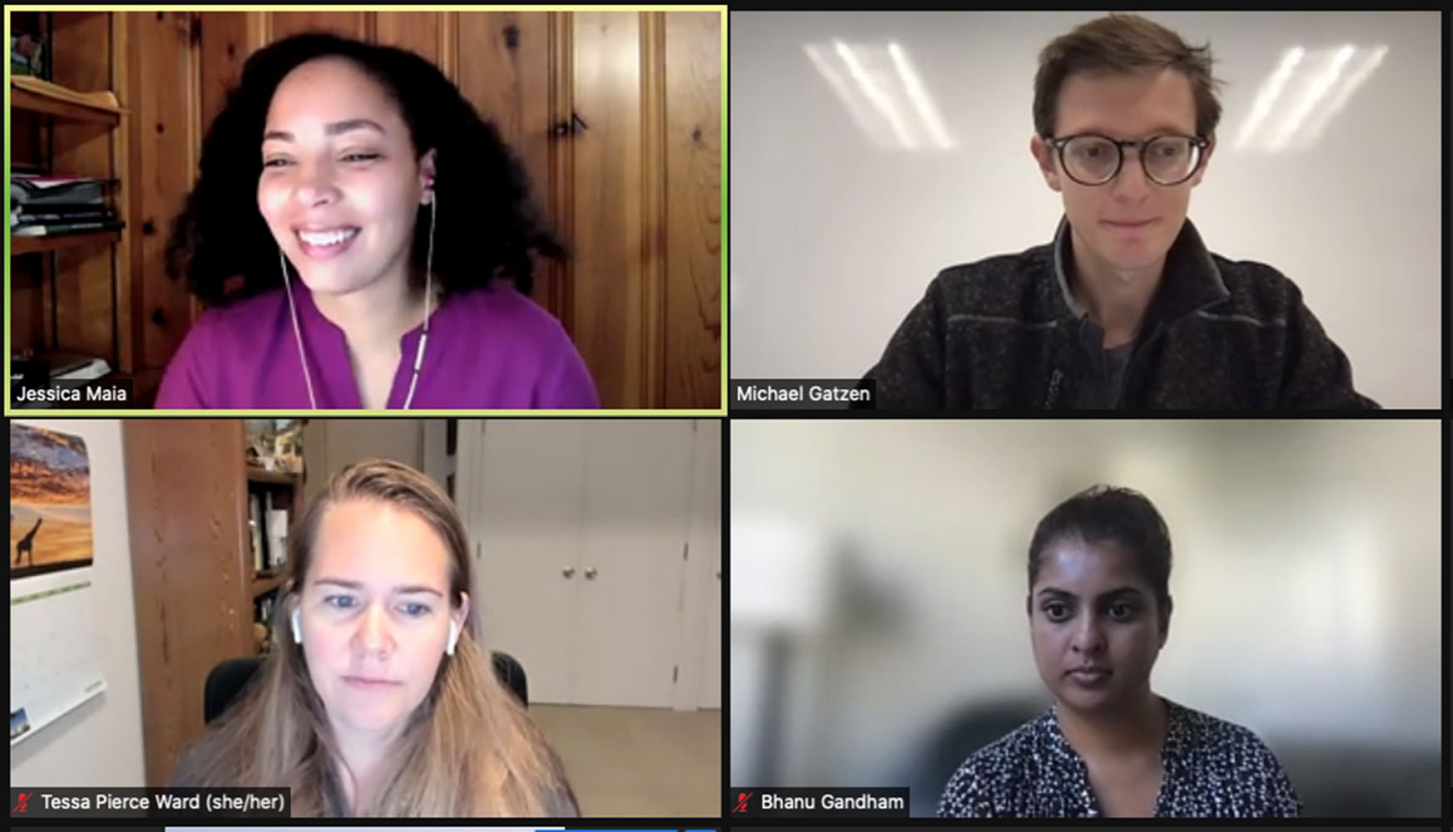
Session chair Jessica Maia and some of the Analysis Tools presenters.

**Figure 8.**
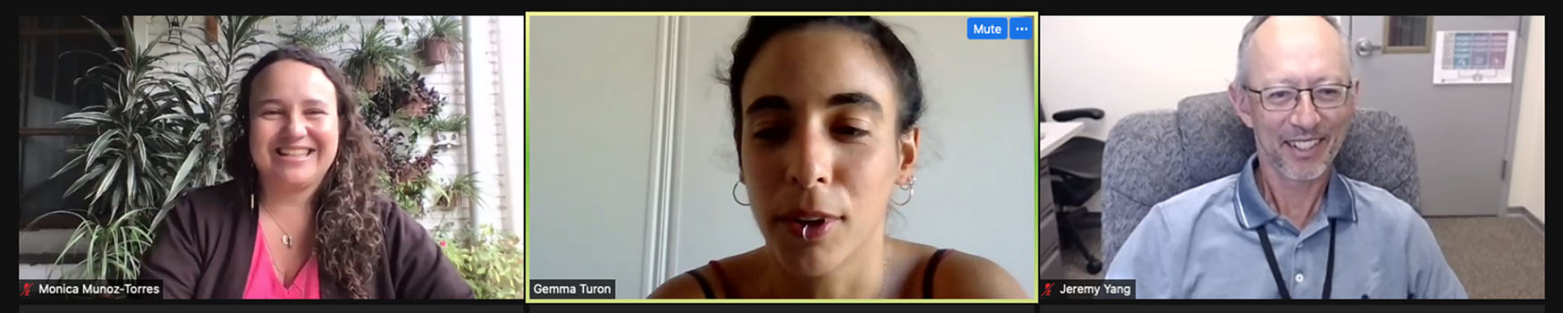
Session chair Monica Munoz-Torres and two of the Translational Bioinformatics session presenters.

Some highlights of the sessions at BOSC 2021:

• BOSC 2021 Chair Nomi Harris, speaking from her home in California at 4:00am local time, opened the conference. The opening session also included an overview of the Open Bioinformatics Foundation (OBF) by OBF President Peter Cock.
• In the Standards and Practices for Open Science session ( Figure 5), speaker Dhrithi Deshpande spoke about the disappointingly low percentage of scientific papers that provide a link to their code (only about 12%—though up from only 1% in 2016!), and noted that articles that share code tend to get cited more.
• The Tools for Open Science session ( Figure 6) started off with a well-received talk by Thorin Tabor about GenePattern, a reproducible research platform built on top of Jupyter Notebook.
• In addition to a keynote talk, the joint session with the Function COSI included a short talk entitled “Completing the functional human proteome together!” by Monique Zahn.
• The Analysis Tools session ( Figure 7) included presentations about a range of new and older tools for performing sequence and structural analyses.
• The session on Workflow Management Systems included updates on widely used workflow systems such as Nextflow, Dockstore and Sapporo, and introductions to newer resources such as WDL Analysis Research Pipelines (WARP) and WorkFlow Package Manager (WFPM).
• A short session on visualization tools and platforms focused on the venerable genome browser, JBrowse, now deployable through Docker and with a major update. It also introduced GO-Figure!, a new viz tool for Gene Ontology terms.
• The Translational Bioinformatics session ( Figure 8) covered hot topics such as knowledge graphs and drug discovery in an open source context, with presentations about the Ersilia Model Hub, Library of Integrated Network-Based Cellular Signatures (LINCS) and Illuminating the Druggable Genome (IDG), and precision oncology using knowledge bases.
• Like last year, BOSC permitted Platinum and Gold sponsors to present short videos without peer review. These short (2-3 minutes) sponsor talks allow our valued sponsors to inform the BOSC community about what they offer.

### Birds of a Feather (BoFs)

**Figure 9.**
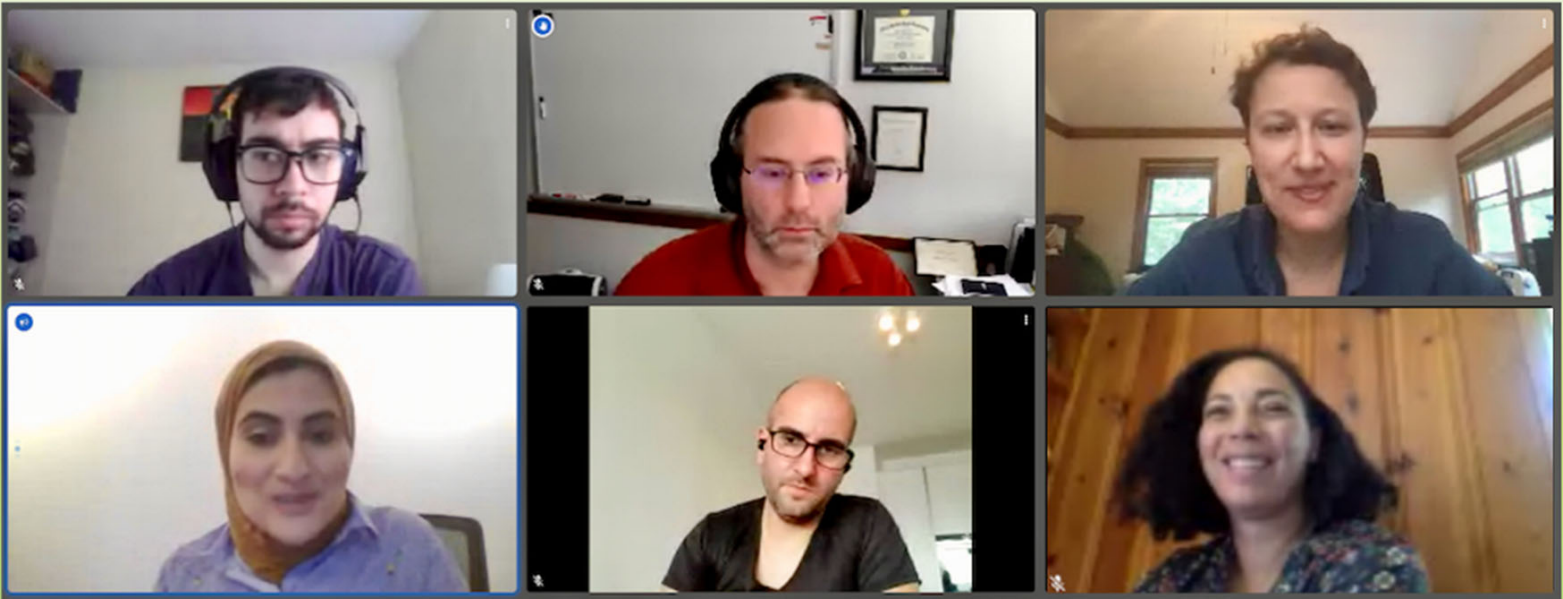
Some of the attendees at one of the 2021 Birds of a Feather sessions.

*Birds of a Feather* are informal, self-organized meetups focused on specific topics, and have long been a popular part of BOSC and ISMB. This year, perhaps due to Zoom fatigue and platform limitations, there were only a handful of BoFs, but the six BOSC-related BoFs fostered some lively dialog (see Figure 9).

The BoF topics this year were:

• New Methods of Programmatic, Web, and Cloud Data Access from National Center for Biotechnology Information (NCBI)
• JBrowse
• Jalview and Friends
• Next steps for computational reproducibility toward fully executable papers
• Interoperability Challenges for Sensitive Biomedical Data

### CoFest

**Figure 10.**
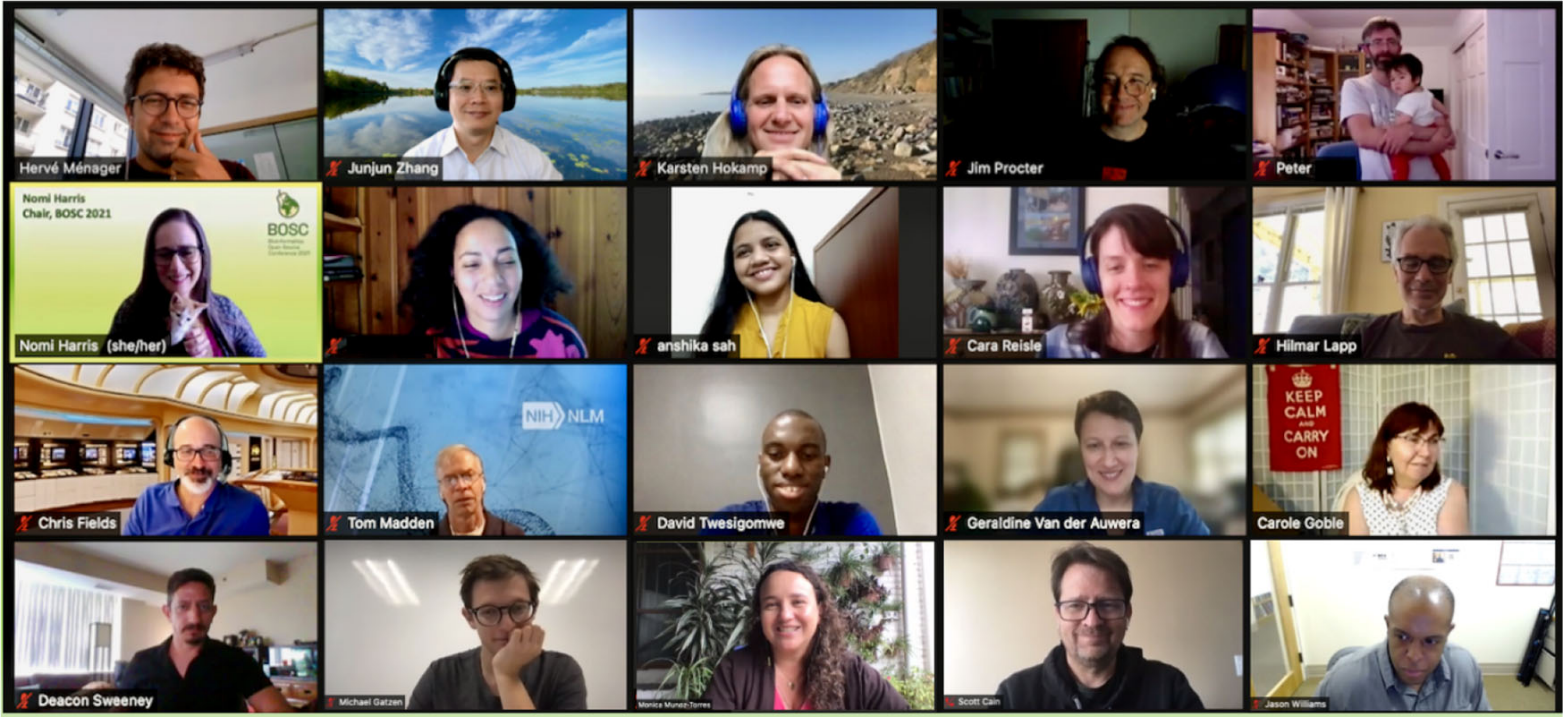
The final BOSC 2021 online “happy hour”.

**Figure 11.**
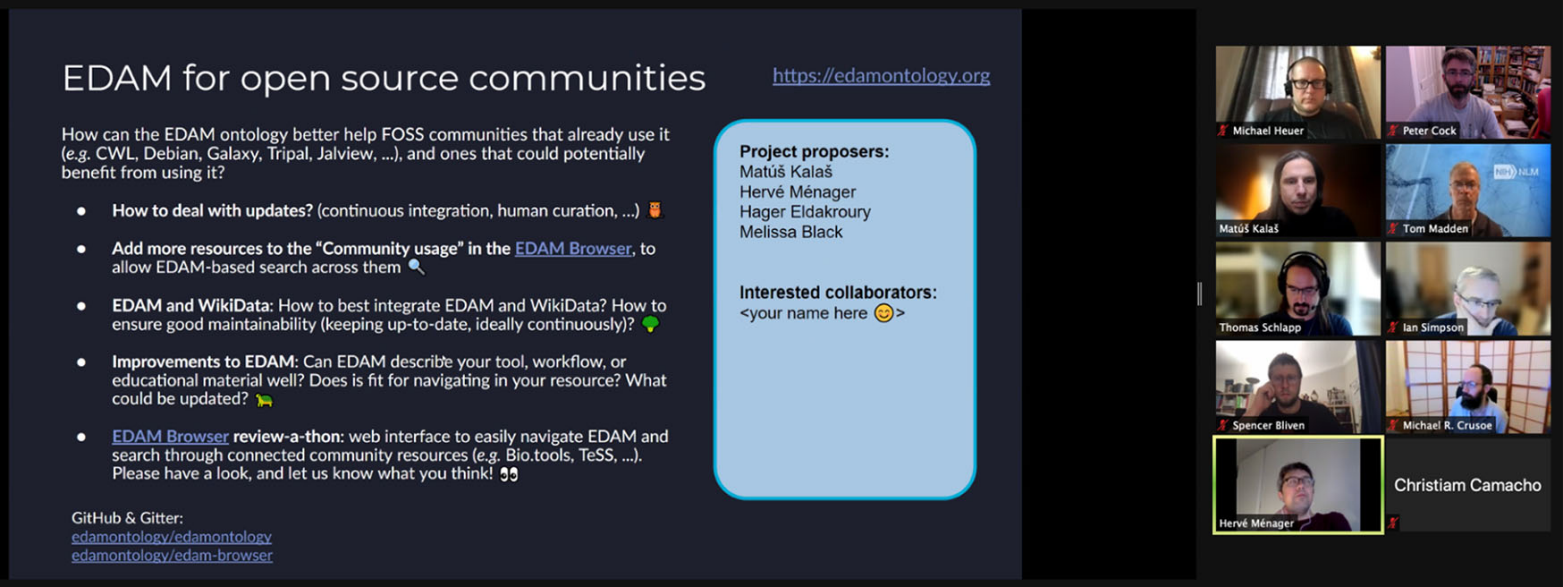
CoFest participants work to make EDAM even more useful to the broader community.

After BOSC/ISMB, which concluded with an online “happy hour” ( Figure 10), there was a two-day CollaborationFest (CoFest). This free, collaborative work event, which has been taking place after BOSC for the last 12 years, was renamed "CollaborationFest" starting in 2018 to acknowledge the importance of activities besides coding, such as working on documentation, training materials, and use cases. In 2020 and again in 2021, CoFest was held online. About 25 people participated in six collaborative projects during the 2021 CoFest ( Figure 11).

## Data availability

No data are associated with this article.

## Consent

All photos in this report are shared under a CC-BY-SA license. All identifiable subjects in the photos were contacted, and they consented to have their photos used in this report.

